# Detection of 8-oxoguanine and apurinic/apyrimidinic sites using a fluorophore-labeled probe with cell-penetrating ability

**DOI:** 10.1186/s12860-019-0236-x

**Published:** 2019-11-27

**Authors:** Dong Min Kang, Jong-Il Shin, Ji Beom Kim, Kyungho Lee, Ji Hyung Chung, Hye-Won Yang, Kil-Nam Kim, Ye Sun Han

**Affiliations:** 1 Department of Advanced Technology Fusion, Konkuk University, 120 Neungdong-ro, Gwangjin-gu, Seoul, 05029 Republic of Korea; 2 Department of Biological Sciences, Konkuk University, Seoul, 05029 South Korea; 3 Department of Applied Bioscience, College of Life Science, CHA University, Pocheon, 11160 South Korea; 4 Department of Marine Life Science, Jeju National University, Jeju, 63243 South Korea; 5 Chuncheon Center, Korea Basic Science Institute (KBSI), Chuncheon, 24341 South Korea

**Keywords:** 8-oxo-7,8-dihydroguanine (8-oxoG), Human ribosomal protein S3 (hRpS3), Transactivator (TAT) proteins, Apurinic/apyrimidinic (AP) site

## Abstract

**Background:**

Reactive oxygen species (ROS) produce different lesions in DNA by ROS-induced DNA damage. Detection and quantification of 8-oxo-7,8-dihydroguanine (8-oxoG) within cells are important for study. Human ribosomal protein S3 (hRpS3) has a high binding affinity to 8-oxoG. In this study, we developed an imaging probe to detect 8-oxoG using a specific peptide from hRpS3. Transactivator (TAT) proteins are known to have cell-penetrating properties. Therefore, we developed a TAT-S3 probe by attaching a TAT peptide to our imaging probe.

**Results:**

A DNA binding assay was conducted to confirm that our probe bound to 8-oxoG and apurinic/apyrimidinic (AP) sites. We confirmed that the TAT-S3 probe localized in the mitochondria, without permeabilization, and fluoresced in H_2_O_2_-treated HeLa cells and zebrafish embryos. Treatment with Mitoquinone (MitoQ), a mitochondria-targeted antioxidant, reduced TAT-S3 probe fluorescence. Additionally, treatment with O8, an inhibitor of OGG1, increased probe fluorescence. A competition assay was conducted with an aldehyde reaction probe (ARP) and methoxyamine (MX) to confirm binding of TAT-S3 to the AP sites. The TAT-S3 probe showed competitive binding to AP sites with ARP and MX.

**Conclusions:**

These results revealed that the TAT-S3 probe successfully detected the presence of 8-oxoG and AP sites in damaged cells. The TAT-S3 probe may have applications for the detection of diseases caused by reactive oxygen species.

## Background

Reactive oxygen species (ROS) are generated by the cellular metabolism or by exogenous factors [1]. 8-Oxo-7,8-dihydroguanine (8-oxoG) is one of the most abundant base lesions generated when DNA is damaged by ROS [1]. 8-OxoG can pair with adenine as well as cytosine, thereby causing G-to-T transversion mutations [2, 3]. This mutation has been associated with the development of cancers in humans [4–6] and must be efficiently removed from DNA to avoid deleterious consequences [7]. Based on previous studies in bacterial cells, base excision repair (BER) has been established as the major pathway for the removal of this lesion [8].

Regardless of the type of damage, the first step in BER is the excision of the damaged base by a glycosylase, which leaves a free ribose sugar, known as the abasic or apurinic/apyrimidinic (AP) site [9]. AP sites are formed following oxidative damage of DNA by ROS [10, 11], and this oxidative damage is associated with cancer, heart disease, Parkinson’s disease, and aging [12, 13]. In humans, human ribosomal protein S3 (hRpS3) exhibits AP lyase activity specific for AP sites in DNA through a beta-elimination reaction [14]. hRpS3 binds tightly to both AP and 8-oxoG sites and physically interacts with proteins known to be involved in repair [15, 16].

8-OxoG and AP sites are the main products of oxidative DNA damage in living organisms [17]. Intracellular and extracellular 8-oxoG levels are regarded as an index of oxidative damage to cells and have been used as a biomarker for a number of diseases, including cancer and aging [18, 19]. Several analytical methods for 8-oxoG and AP sites have been established, but more efficient detection methods are needed. Therefore, methods that can be used to directly, selectively, precisely, and rapidly detect 8-oxoG in cells would be useful for evaluating intracellular oxidative stress and DNA damage [20, 21].

The intersection of molecular imaging and site-specific peptide chemistry has resulted in the generation of highly efficient and stable peptide probes for different imaging modalities, and the synthesis of peptide probes has attracted much attention [22–24]. Therefore, we decided to develop a probe based on hRpS3, which has specific and high binding affinity for DNA lesions. Additionally, in order for the probe to be visualized, it must pass through the cellular membrane. Although small molecules are able to cross the cellular membrane independently, many larger molecules cannot owing to their physicochemical characteristics [25]. A delivery system must be efficient, safe, and nontoxic. The transactivator (TAT) domain (11 amino acids, YGRKKRRQRRR) of the human immunodeficiency virus-1 (HIV-1) TAT protein can efficiently deliver proteins into cells and appears to not be limited by the size of the fusion protein [26]. Therefore, we bound a TAT peptide to an S3 peptide using a GG linker.

Mitochondria are the major consumers of cellular oxygen and, therefore, play a central role in ROS biology. Incomplete processing of oxygen and/or release of free electrons results in the production of oxygen radicals. Under normal physiological conditions, a small fraction of oxygen consumed by mitochondria is converted to superoxide anions, H_2_O_2_, and other ROS [27]. Mitochondria have their own ROS scavenging mechanisms that are required for cell survival [28]. It has been shown, however, that mitochondria produce ROS at a rate higher than their scavenging capacity, resulting in the incomplete metabolism of approximately 1–3% of consumed oxygen [29, 30]. The byproducts of incomplete oxygen metabolism are superoxide, H_2_O_2_, and hydroxyl radicals. In the presence of reduced transition metals, H_2_O_2_ can produce the highly reactive OH•, which can cause extensive damage to DNA, proteins, and lipids. ROS-induced mitochondrial DNA damage can lead to mitochondrial dysfunction, and it is, therefore, important to properly detect mitochondrial DNA damage. The roles of mitochondria in energy production and programmed cell death make this organelle a prime target for the treatment of several disease states [8, 31]. The TAT-S3 probe targeting mitochondria may, thus, be suitable for therapeutic studies focused on mitochondria.

Zebrafish have been traditionally used in the fields of molecular genetics and developmental biology as a model organism for drug discovery and toxicological studies because of their physiological similarity to mammals [32–35]. Moreover, zebrafish have been used as a model for human disease and development [36].

In previous studies, we generated an hRpS3-peptide probe that could be used to detect 8-oxoG via a fluorescence assay [37]. We generated a new probe for 8-oxoG and AP sites consisting of TAT peptide and hRpS3, termed TAT-S3. The TAT-S3 probe targets ROS-induced mitochondrial damage and has the ability to penetrate cells. In this study, we report the development of this new and highly sensitive TAT-S3 probe for the detection of 8-oxoG and AP sites.

## Results

### Synthesis of the TAT-S3 probe

**Fig. 1:**
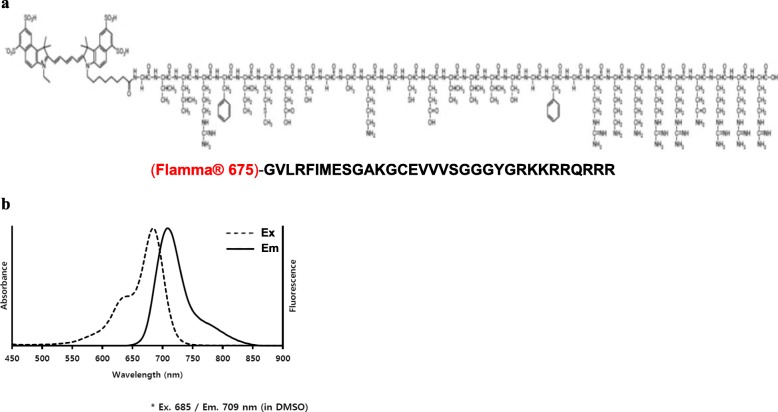
Structure and spectrum of the TAT-S3 probe. **a** Chemical Structure of the TAT-S3 probe. **b** Normalized absorbance and fluorescence emission spectra for the TAT-S3 probe

We previously developed an imaging probe to detect 8-oxoG using a specific peptide of hRpS3 [37]. A TAT (47–57, YGRKKRRQRRR) peptide that can penetrate cells was attached at the C-terminus of the S3 probe, and a two-amino-acid GG linker was added between the S3 probe and TAT to generate the new TAT-S3 probe. The ability of the TAT-S3 probe to bind 8-oxoG was similar to that of the S3 probe. The TAT-S3 probe was labeled with a fluorescent dye (Flamma-675) at the amine of the N-terminal glycine for visualization (Fig. 1a). The basic spectroscopic properties of the TAT-S3 probe were assessed in vitro (DMSO), revealing an absorption band at 685 nm and an emission band at 709 nm (Fig. 1b).

### Effects of the TAT-S3 probe on cell viability

**Fig. 2:**
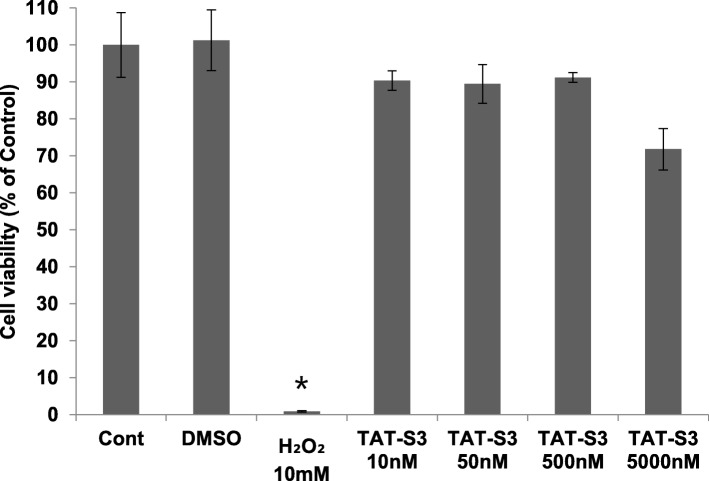
Cell viability assay of the TAT-S3 probe on HeLa cells. HeLa cells were treated with the indicated concentrations of the TAT-S3 probe for 24 h. Cell viability was determined using an MTT assay. Significant differences are indicated as *p < 0.01. Data shown represent the means of 3 individual experiments. * p < 0.01 compared with untreated group

The cytotoxicity of the TAT-S3 probe was evaluated in HeLa cells via 3-(4,5-dimethylthiazol-2-yl)-2,5-diphenyl-2 tetrazolium bromide (MTT) assay. HeLa cells were treated with the TAT-S3 probe and compared with untreated cells. Various concentrations of the TAT-S3 probe were chosen for analysis (0, 5, 500, and 5000 nM) (Fig. 2), and cell viability was analyzed after 24 h of incubation with the TAT-S3 probe. Treatment with H_2_O_2_ (10 mM) was chosen as a positive control and caused a viability decrease of 99% compared with that of untreated cells. The pobe did not show any toxic effects at most concentrations. However, at the highest concentration (5000 nM), cell viability was reduced to 71%.

### Binding of the TAT-S3 probe to substrate containing 8-oxoG and AP sites

**Fig. 3:**
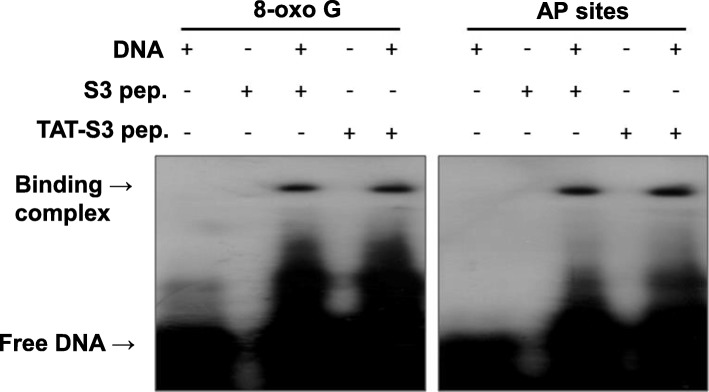
DNA binding assay of the TAT-S3 and S3 probe for the DNA containing 8-oxoG or apurine/apyrimidine. The DNA-protein complex was incubated for 2 h at 37 °C with 20 pmol each of the TAT-S3 and S3 probes, and a 5 ‘end-radiolabeled 39 mer nucleotide duplex (7 pmol) containing either 8-oxoG or AP sites

In a previous study, we confirmed that an S3 probe containing a K132 (K134 of *Drosophila melanogaster* RpS3) amino acid residue, which plays a crucial role in the binding of hrpS3 and 8-oxoG, specifically binds to substrates containing 8-oxoG [37]. In addition to 8-oxoG, hRpS3 is known to have binding affinity to AP sites, and K132 amino acid residues are known to play an important role in specific binding to AP sites [38, 39]. We performed a DNA-peptide binding assay to determine if the S3 probe from hRpS3 containing a K132 amino acid residue could specifically bind to AP sites and confirmed that the TAT-S3 probe specifically binds to substrates containing AP sites. To determine whether the TAT peptide affects the ability of the TAT-S3 probe to bind to specific substrates, we performed DNA binding assays to confirm binding of the TAT-S3 probe to 8-oxoG and AP sites (Fig. 3). We confirmed that the TAT-S3 probe, similarly to the S3 peptide probe, specifically binds to damaged DNA substrates including 8-oxoG or AP sites, and the band intensities of the two peptides are the same. These results showed that the TAT-S3 probe binds to 8-oxoG and AP sites with an affinity similar to that of the S3 peptide probe.

### Cellular uptake and localization of TAT-S3 probe

**Fig. 4:**
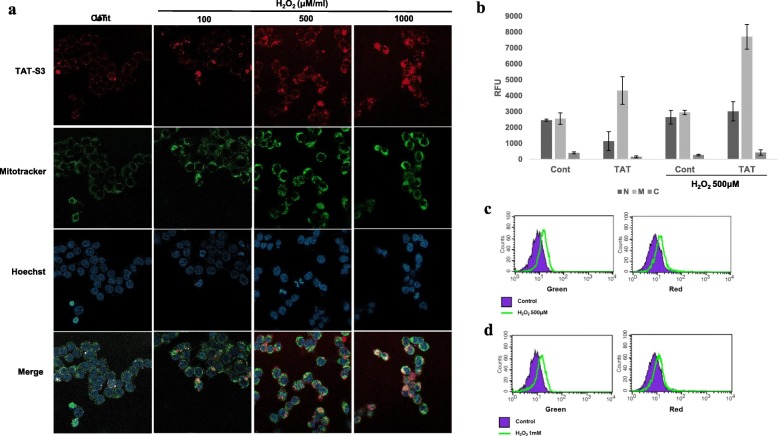
Cellular localization and uptake of the TAT-S3 probe. **a** HeLa cells were treated with the TAT-S3 probe for 24 h and then treated with 100, 500, and 1000 μM H_2_O_2_ for 1 h. HeLa cells were stained with Mitotracker (Green) at 37 °C for 15 min and then stained with Hoechst (Blue) at 37 °C for 5 min. The staining intensity was observed by confocal laser scanning microscope. **b** HeLa cells were treated the TAT-S3 probe (2 μM) for 24 h and then treated with 500 μM H_2_O_2_ for 1 h. HeLa cells were sonicated and centrifuged to obtain three fractions: Nuclear (N), Mitochondrial (M) and Cytoplasmic (**c**). Fluorescence was measured on a fluorescence microplate reader at 685/709 nm. Data shown represent the means of 3 individual experiments. **c** HeLa cells were treated with the TAT-S3 probe for 24 h and then treated with 500 μM H_2_O_2_ for 1 h. HeLa cells were stained with Mitotracker at 37 °C for 15 min. The intensities of the TAT-S3 probe (Red) and Mitotracker (Green) were detected using flow cytometry. **d** HeLa cells were treated with the TAT-S3 probe for 24 h and then treated with 1000 μM H_2_O_2_ for 1 h. HeLa cells were stained with Mitotracker at 37 °C for 15 min. The intensities of the TAT-S3 probe (Red) and Mitotracker (Green) were detected using flow cytometry

**Fig. 5:**
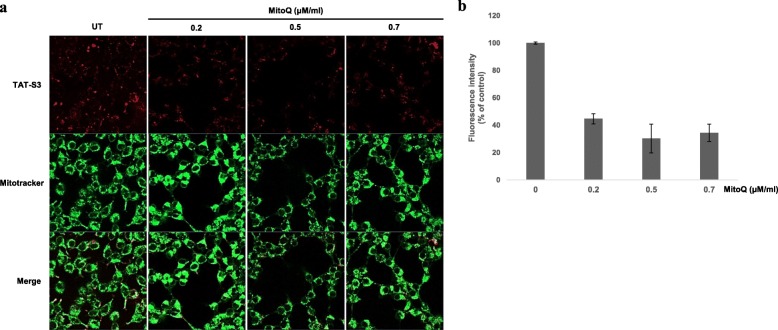
Decrease of fluorescence intensity by MitoQ in ROS-induced mitochondria DNA damage. **a** HeLa cells were treated with the TAT-S3 probe for 24 h. HeLa cells were treated with 0.2, 0.5 and 0.7 μM MitoQ for 30 min and then treated with 500 μM H_2_O_2_ for 1 h. HeLa cells were stained with Mitotracker (Green) at 37 °C for 15 min. **b** Fluorescence intensity of the TAT-S3 probe in (**a**) was measured by ImageJ software. Data shown represent means of 3 individual experiments

**Fig. 6:**
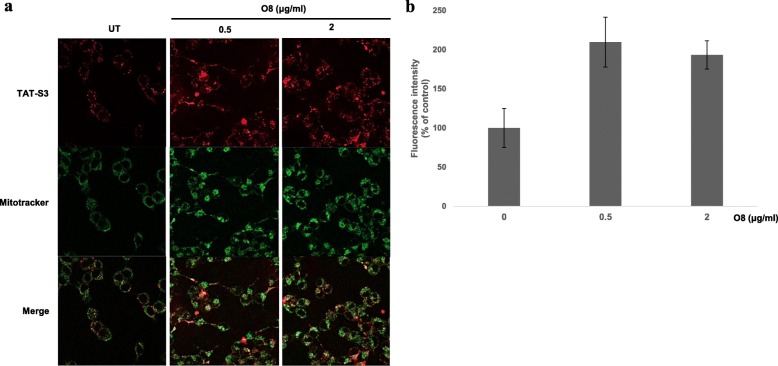
Increase of fluorescence intensity by O8 in HeLa cells. **a** HeLa cells were treated with the TAT-S3 probe at 37 °C for 24 h. HeLa cells were treated with 0.5 and 2 μg O8 for 1 h and then treated with 500 μM H_2_O_2_ for 1 h. HeLa cells were stained with Mitotracker (Green) at 37 °C for 15 min. **b** Fluorescence intensity of the TAT-S3 peptide probe in (**a**) was measured by ImageJ software. Data shown represent means of 3 individual experiments

To demonstrate that the TAT-S3 probe has the ability to penetrate cells, HeLa cells were treated with the TAT-S3 probe (100 nM), incubated for 24 h at 37 °C, and imaged using confocal microscopy. To verify the ability of the peptide to target the nucleus and mitochondria, Hoechst blue and MitoTracker green fluorescent dyes were used. The merged images showed localization of Hoechst, MitoTracker, and the TAT-S3 probe labeled with Flamma 675 (Fig. 4a). The TAT-S3 probe was not localized in the nucleus. However, the TAT-S3 probe was localized in the mitochondria. We confirmed the localization of the probe in the nucleus, mitochondria, and cytoplasm through cell fractionation. HeLa cells were treated with 500 μM H_2_O_2_ to induce DNA damage and then treated with 2 μM TAT-S3 probe. After sonication and centrifugation, the various fractions were obtained. The fluorescence from each fraction was measured at 685/709 nm on a fluorescence plate reader. We found that more than 90% of the fluorescence of the TAT-S3 probe was present in the mitochondrial fractions in the H_2_O_2_-treated HeLa cells (Fig. 4b). The level of the TAT-S3 probe in mitochondria was 2.5 times higher than that in the nucleus, suggesting that the TAT-S3 probe specifically binds to damaged DNA in mitochondria. Levels of the TAT-S3 probe were 1.8 times higher in mitochondria treated with H_2_O_2_ than in untreated mitochondria. In addition, flow cytometry results showed that the TAT-S3 probe and MitoTracker were colocalized (Fig. 4c, d). We next determined whether the TAT-S3 probe could be used to detect the reduction in mitochondrial damage by ROS using Mitoquinone (MitoQ). The fluorescence intensity was decreased dose-dependently by MitoQ by about 60% (Fig. 5a, b). These results indicated that the TAT-S3 probe can be used to detect the reduction in ROS-induced mitochondrial damage by MitoQ. In addition, we evaluated whether the TAT-S3 probe can be used to detect the increase in 8-oxoG in DNA damaged by ROS using O8, an OGG1 inhibitor. The fluorescence intensity was increased dose-dependently by O8 by about 2 times (Fig. 6a, b). These results confirmed that the TAT-S3 probe can be used to detect increases in 8-oxoG induced by O8.

### Comparison of the binding of the TAT-S3 probe, methoxyamine (MX), and aldehyde reaction probe (ARP) to AP sites

**Fig. 7:**
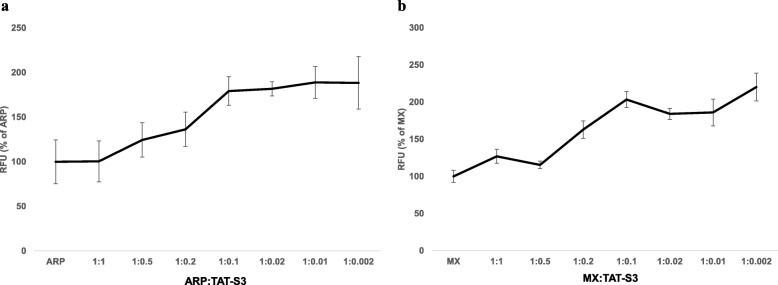
Competition assay of the TAT-S3 probe and ARP or MX. **a** ARP or **b** MX for AP sites in genomic DNA treated with H_2_O_2_ (500 μM) for 24 h. The ratios of ARP:TAT-S3 peptide probe used in this study were: 0, 0.5, 0.2, 0.1, 0.02, 0.01, 0.002. The ratios of MX:TAT-S3 peptide probe used in this study were: 0, 0.5, 0.2, 0.1, 0.02, 0.01, 0.002. Data shown represent the means of 3 individual experiments

We performed a competition assay of the TAT-S3 probe and either ARP or MX. ARP and MX are commonly used to evaluate AP sites. Using ARP and MX, the binding affinity of the TAT-S3 probe for AP sites was measured. Keeping the molar concentration of the TAT-S3 probe constant, the molar concentrations of ARP/MX were adjusted so that the MX:TAT-S3 or ARP:TAT-S3 ratio ranged from 0.5 to 0.002. These solutions of MX or ARP and the TAT-S3 probe were added to DNA for 24 h at 37 °C. Fluorescence was measured following ethanol precipitation to remove unbound probes. The results indicated that the TAT-S3 probe competitively binds to AP sites in the presence of ARP/MX (Fig. 7). The binding ability of the TAT-S3 probe for AP sites was 1.2 times higher than that of ARP when the TAT-S3 probe concentration was 2 times higher than that of ARP (Fig. 7a) and 1.6 times higher than that of MX when the TAT-S3 probe concentration was 5 times higher than that of MX (Fig. 7b). Although the AP site-binding ability of the TAT-S3 probe was slightly lower at equivalent concentrations of ARP/MX, these results confirmed that the TAT-S3 probe binds to AP sites, which is consistent with our results from the DNA binding assay.

### Cellular ATP levels

**Fig. 8:**
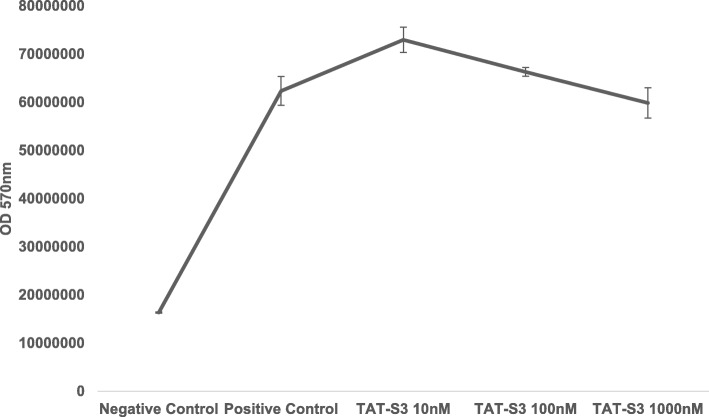
ATP assay of the TAT-S3 probe on HeLa cells. HeLa cells were treated with the indicated concentrations of the TAT-S3 probe for 24 h. Cells were collected and lysed with 0.5% TCA. ATP levels were analyzed for luciferase activity using a luminometer. Data shown represent the means of 3 individual experiments

To determine the effect of the TAT-S3 probe in mitochondria, an ATP assay was performed. Various concentrations of the TAT-S3 probe (10 nM, 100 nM, and 1000 nM) were added to cultured HeLa cells, and the luminescence intensity of firefly luciferin-luciferase was measured. ATP levels in cells treated with different concentrations of the TAT-S3 probe were approximately the same as those in untreated cells (Fig. 8). These results confirmed that the TAT-S3 probe did not affect mitochondrial function.

### Effect of the TAT-S3 probe in the zebrafish model

**Fig. 9:**
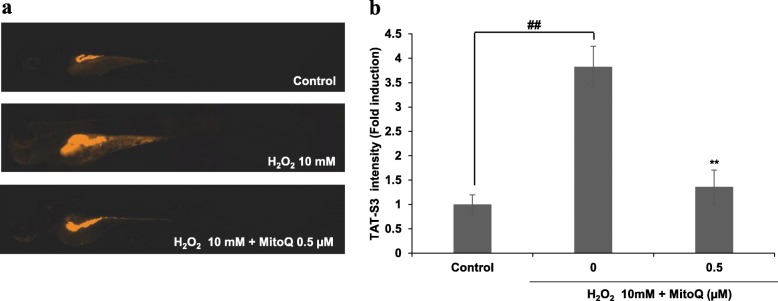
The inhibitory effect of MitoQ on H_2_O_2_-induced oxidative stress in zebrafish. **a** The zebrafish were pre-treated with 0.5 μM of MitoQ and then stimulated with 10 mM H_2_O_2_. **b** The intensity was analyzed after treatment with the TAT-S3 peptide probe using Image J. Experiments were performed in a triplicate and data is presented as the mean ± SE. **p < 0.01 compared with H_2_O_2_ treated group. ##*p* < 0.01 compared with untreated group

To assess whether changes in fluorescence were influenced by the TAT-S3 probe in an in vivo model, we used an H_2_O_2_-induced oxidative stress zebrafish model. The fluorescence intensity of larvae was analyzed after treatment with the TAT-S3 probe. As shown in Fig. 9, the fluorescence intensity was significantly increased by H_2_O_2_ treatment compared with the control. However, treatment of the zebrafish with MitoQ significantly decreased the fluorescence intensity. This result suggested that the zebrafish model is suitable for in vivo evaluation of changes in fluorescence of the TAT-S3 probe. Further, MitoQ was shown to be suitable as a positive control that can decrease oxidative stress in the zebrafish model.

## Discussion

Our TAT-S3 probe was capable of targeting mitochondrial DNA damage. Mitochondrial ROS are generated as normal byproducts of oxidative metabolism. Approximately 3% of the consumed mitochondrial oxygen is not completely reduced [40], and leaky electrons can easily interact with molecular oxygen to generate ROS, such as superoxide anions [41]. Oxidative stress is a conserved signal for cell death and is involved in a variety of cell death paradigms. Hence, small molecules such as ROS can affect the complex networks of proteins mediating the induction and execution of cell death. Mitochondrial impairment results in overproduction of ROS, promoting the onset of diseases characterized by various clinical symptoms.

hRpS3 is a remarkably versatile protein involved in DNA repair, cell death, inflammation, tumorigenesis, and transcriptional regulation [38, 42]. Besides its role in the maturation of ribosomes, hRpS3 participates in DNA repair [43]. hRpS3 cannot remove 8-oxoG from damaged DNA but does have a high binding affinity for 8-oxoG. According to Hegde et al., lysine K32 of hRpS3 is required for binding to DNA containing 8-oxoG [44].

The development of peptide drugs and therapeutic proteins is limited by the selectivity of the cell membrane, which results in poor permeability of these compounds [39]. Many pharmaceutical agents are delivered intracellularly to exert their therapeutic effects inside the cytoplasm or on individual organelles, such as the nuclei (for gene and antisense therapy), lysosomes (for the delivery of deficient lysosomal enzymes), and mitochondria (for pro-apoptotic anticancer drug delivery) [45]. The TAT protein from HIV-1 is able to deliver biologically active proteins in vivo and has generated considerable interest for use in protein therapeutics [46–51]. Therefore, we used a TAT peptide for the delivery of our S3 probe into cells.

We developed the TAT-S3 probe using a TAT peptide and Flamma 675 attached to a specific peptide of hRpS3 (Fig. 1). The TAT-S3 probe was not toxic (Fig. 2) and had a similar binding ability to 8-oxoG and AP sites as compared to that of the S3 peptide alone (Fig. 3). When cell damage was increased by H_2_O_2_, the fluorescence intensity of the TAT-S3 probe increased and was localized to the mitochondria (Fig. 4a). In cell fractionation studies, the TAT-S3 probe was highly localized in the mitochondria (Fig. 4b), while in flow cytometry, the fluorescence of the TAT-S3 probe and MitoTracker was colocalized (Fig. 4c, d). The fluorescence intensity of the TAT-S3 probe was decreased by treatment with MitoQ (Fig. 5). On the other hand, the fluorescence intensity of the TAT-S3 probe was increased by treatment with O8 (Fig. 6). These results indicated that the TAT-S3 probe is sensitive to mitochondrial DNA damage. Therefore, the TAT-S3 probe could be used to determine therapeutic effects in studies of mitochondrial damage. The binding of the TAT-S3 probe to AP sites was weaker than that of ARP/MX (Fig. 7), but this effect could be compensated for by increased concentrations of the TAT-S3 probe. These results confirmed that the TAT-S3 peptide probe competitively binds to AP sites. Cellular ATP levels were not altered by treatment with the TAT-S3 probe (Fig. 8), suggesting that treatment with the probe did not alter mitochondrial function. As an animal model, zebrafish have been widely used in studies on molecular genetics, developmental biology, drug discovery, and toxicology because of their physiological similarity to mammals [52–54]. Therefore, we evaluated the effect of MitoQ on the fluorescence intensity of the TAT-S3 probe by using an H_2_O_2_-induced oxidative stress zebrafish model, in which the fluorescence intensity of the TAT-S3 probe was decreased by treatment with MitoQ (Fig. 9).

## Conclusion

In conclusion, we have developed a novel imaging probe for 8-oxoG and AP sites utilizing an hRpS3 peptide that specifically detects 8-oxoG and AP sites in HeLa cells without permeabilization. The TAT-S3 probe can distinguish 8-oxoG and AP sites from other nucleosides. Fluorescence of the TAT-S3 probe was observed in cells 24 h after treatment. The TAT-S3 probe was not easily degraded intracellularly and retained its ability to detect 8-oxoG and AP sites. Fluorescence of the TAT-S3 probe was observed 36 h after treatment. Studies using microscopy and isolated mitochondria indicated that the peptide was taken up by the mitochondria. In zebrafish, the TAT-S3 probe was found to specifically bind to mitochondria. Thus, the TAT-S3 probe could be useful as a probe for detecting mitochondrial DNA damage, which could be advantageous in the development of therapeutics targeting mitochondria.

## Methods

### Peptide synthesis

Peptides were synthesized according to the methods described by Han et al. [37]. The peptides were labeled in dye (FPR-675; BioActs, Incheon, South Korea) at the amine of the N-terminal glycine. The peptides were combined with the TAT peptide at the amine of the C-terminus using a GG linker.

### Cell culture

HeLa cells were obtained from the Department of Biological Sciences of Konkuk University (Seoul, Korea). HeLa cells were grown in Dulbecco’s Modified Eagle medium (DMEM; Welgene, Gyeongsan, South Korea) containing 10% fetal bovine serum (Biowest, Nuaillé, France) and 1% penicillin-streptomycin solution (Sigma-Aldrich) at 37 °C in a 5% CO_2_ incubator.

### MTT assay

MTT was purchased from Sigma-Aldrich (98% purity). HeLa cells were seeded in 24-well plates at a density of 1 × 10^5^ cells per well and incubated for 24 h. The cells were treated with the TAT-S3 probe at different concentrations and incubated for 24 h in complete medium. Cells treated with 10 mM H_2_O_2_ for 1 h were used as a positive control. MTT solution (0.5 mg/mL) was added to each well and incubated for 1 h, and then DMSO was added and incubated for 5 min. Absorbance was measured at 570 nm on a microplate reader.

### DNA binding assay of the TAT-S3 probe and S3 probe

A DNA binding assay was conducted using a 5′-end-labeled DNA oligonucleotide duplex containing either a single AP site (AP-39mer) or a single 8-oxoG residue (8-oxoG-39mer). The DNA binding assay was performed in reaction buffer (30 mM KCl, 30 mM HEPES, pH 7.4, and 0.01% Triton X-100) with 2 μM of the TAT-S3 and S3 probes. The radiolabeled 39-mer oligonucleotide duplex (7 pmol) was immediately added to the TAT-S3 and S3 probes. After incubation at 37 °C for 3 h, reactions were terminated using 6× DNA loading dye (Bio Basic Inc., Markham, ON, Canada). Samples were loaded on a 10% nondenaturing polyacrylamide gel in 1× TBE buffer (450 mM tris, 450 mM boric acid, 1 mM EDTA, pH 8.0). After electrophoresis, gels were vacuum dried and suctioned.

### Confocal fluorescence microscopy

Digital images were obtained with a super-resolution confocal laser scanning microscope (LSM 800, Carl Zeiss, Oberkochen, Germany). HeLa cells were seeded in a confocal dish at a density of 2 × 10^5^ cells and treated with 100 nM TAT-S3 probe in complete medium. After 24 h, the cells were treated with 500 μM H_2_O_2_. After 1 h, the cells were triple washed with PBS and stained with Hoechst (1:5000) for 10 min. After an additional three washes with PBS, MitoTracker Green FM (Invitrogen, Carlsbad, CA, USA) was added at a final concentration of 20 nM and incubated for 15 min. The cells were again washed three times with PBS. A blue pseudocolor was applied to visualize the nuclear stain, a green pseudocolor was applied to visualize the mitochondrial stain, and a red pseudocolor was applied to visualize the localization of the TAT-S3 probe within cells.

### Cell fractionation

HeLa cells were seeded in 1 × 10^5^ cells/60-mm^2^ cell culture dish. The cells were treated with 2 μM TAT-S3 probe and incubated for 24 h. Hoechst was added at a final dilution of 1:5000. The cells were washed three times with PBS and then collected and lysed with a sonicator. The lysate was centrifuged at 300×g for 5 min. The supernatant was a cell-free extract, and the pellet was resuspended with 200 μL of PBS and centrifuged at 600×g for 10 min. This supernatant contained nuclei, and the pellet was resuspended with 200 μL of PBS and centrifuged at 16,000×g for 30 min. The final pellet contained mitochondria and was resuspended with 200 μL of PBS.

### Genomic DNA competition assay with ARP/MX and the TAT-S3 probe

Genomic DNA was extracted using a genomic DNA extraction kit (Bioneer, Daejeon). Solutions of ARP/MX and the TAT-S3 probe were prepared in H_2_O; the ARP/MX concentration was adjusted to 0–10 μM while maintaining the TAT-S3 probe concentration at 10 μM. The ratios of ARP/MX:TAT-S3 probe used in this study were as follows: 0.002, 0.01, 0.02, 0.1, 0.2, and 0.5. The samples were prepared in triplicate by adding 10 μL ARP or MX/TAT-S3 solution to 5 μL genomic DNA (100 μg/mL). Samples were incubated at 37 °C for 24 h in the dark. Tris-EDTA buffer (85 μL, pH 7.0; Sigma-Aldrich) and 1 μL of glycogen (Sigma-Aldrich) were added to the samples, followed by 10 μL of 3 M sodium acetate. Ice-cold ethanol (300 μL) was added, and the DNA was purified by ethanol precipitation. The pellet was washed three times with 70% ethanol and dissolved in 100 μL H_2_O. Samples were added to a 96-well black plate (Corning, Corning, NY, USA) and analyzed at 685 nm excitation and 709 nm emission.

### Flow cytometry

HeLa cells were seeded in 3 × 10^5^ cells/60 mm^2^ dish and cultured. The cells were treated with the TAT-S3 probe (100 nM) for 24 h, followed by the indicated concentration of H_2_O_2_ for 1 h. After washing three times with PBS, the cells were treated with 20 nM MitoTracker Green FM for 15 min and then washed three times with PBS. The cells were collected with a scraper, and the cell lysate (100 μL) was analyzed via flow cytometry (CytoFLEX, Beckman Coulter, Brea, CA, USA).

### ATP assay

ATP assays were performed according to the manufacturer’s protocol (ATP assay kit, Promega, Madison, WI, USA), and detection was performed with a luminometer (Veritas™, Santa Clara, CA, USA). HeLa cells were seeded in 3 × 10^5^ cells/60-mm^2^ dish. The cells were treated with the TAT-S3 probe, incubated for 24 h, and then collected and lysed with 0.5% trichloroacetic acid solution (TCA, Sigma-Aldrich). The lysate was mixed with Tris-EDTA buffer, pH 8.0 (Sigma-Aldrich), and then 100 μL of cell lysate was analyzed for luciferase activity using a luminometer.

### Measurement of antioxidant effects by confocal microscopy

HeLa cells were seeded in confocal dish at a density of 2 × 10^5^ cells and treated with 100 nM TAT-S3 probe. After 24 h, the cells were treated with 0.5 μg/mL MitoQ (BioVision, Milpitas, CA, USA), a mitochondria-targeted antioxidant. After 1 h, the cells were washed three times with PBS, treated with 20 nM MitoTracker Green FM for 15 min, and washed again three times with PBS. Green pseudocolor was applied to visualize mitochondrial staining, while red pseudocolor was applied to visualize the localization of the TAT-S3 probe within cells.

### Measurement of effects of OGG1 inhibitor by confocal microscopy

HeLa cells were in confocal dish at a density of 2 × 10^5^ cells and treated with the TAT-S3 probe (100 nM) alone or in combination with O8 (Sigma-Aldrich), an inhibitor of OGG1. After 24 h, the cells were washed three times with PBS, treated with 20 nM MitoTracker Green FM for 15 min, and washed again three times with PBS. Green pseudocolor was applied to visualize mitochondrial staining, while red pseudocolor was applied to visualize the localization of the TAT-S3 probe within cells.

### Maintenance of zebrafish

Zebrafish were purchased from a commercial dealer (Seoul aquarium, Seoul, Korea) and maintained and bred according to the methods described by Kim et al. [55]. All animal experiments were approved by the Jeju National University Animal Care and Use Committee (2016–0052).

### Measurement of antioxidant effect in zebrafish embryos

From approximately 7–9 h post-fertilization (hpf), 15 embryos were transferred to individual wells of a 12-well plate containing 1.8 mL embryo medium. The embryos were treated with 0.5 μM MitoQ. After 1 h, 10 mM H_2_O_2_ was added to the embryos exposed to MitoQ for up to 72 hpf. Then, zebrafish larvae at 72 hpf were individually transferred to a 96-well plate, treated with 100 nM TAT-S3 probe, and incubated for 24 h in the dark at 28.5 ± 0.5 °C. The zebrafish larvae were rinsed three times with fresh embryo medium. After anesthesia with 0.03% MS-222, stained larvae were observed and photographed under a microscope (Gen 5 version 3.03, BioTek, Winooski, VT, USA). The fluorescence intensity of larvae was quantified using the ImageJ program.

### Statistical analysis

The values in this study are representative of at least three independent experiments. All results are shown as the means ± S.D. Statistical analysis of the data between experimental groups was performed using Student’s t-test. *P* values less than 0.05 were considered statistically significant.

## Data Availability

All data generated or analyzed during this study are included in this article and its supplementary information files.

## Abbreviations

8-oxoG: 8-oxo-7,8-dihydroguanine
AP: Apurinic/apyrimidinic
ARP: Aldehyde reaction probe
BER: Base excision repair
HIV-1: Human immunodeficiency virus-1
hpf: Hours post-fertilization
hRpS3: Human ribosomal protein S3
MitoQ: Mitoquinone
MTT: 3-(4,5-dimethylthiazol-2-yl)-2,5-diphenyl-2 tetrazolium bromide
MX: Methoxyamine
ROS: Reactive oxygen species
TAT: Transactivator
